# Right Ventricular Dimensions and Tricuspid Annular Plane Systolic Excursion among Medical Students of Tertiary Care Hospital: A Descriptive Cross-sectional Study

**DOI:** 10.31729/jnma.5302

**Published:** 2020-09-30

**Authors:** Nimesh Poudel, Mahesh Bhattarai, Laxmi Raj Bhatt, Dambar Bahadur Karki

**Affiliations:** 1 Department of Internal Medicine, Kathmandu Medical College and Teaching Hospital, Sinamangal, Kathmandu, Nepal; 2 Department of Cardiology, Kathmandu Medical College and Teaching Hospital, Sinamangal, Kathmandu, Nepal

**Keywords:** *echocardiography*, *heart*, *right ventricle*

## Abstract

**Introduction:**

The change in morphology and functions of the right ventricle is an important predictor of heart and lung disease. There is limited data on the normal dimension of the right ventricle. The study aimed to find the right ventricular diameter, its thickness, and tricuspid annular plane systolic excursion in healthy male medical students of a tertiary care hospital.

**Methods:**

It is a descriptive cross-sectional study conducted in healthy medical students of Kathmandu Medical College and Teaching Hospital, from February-April, 2019. Ethical approval was taken from the institutional review committee (reference number 120720193). Convenient sampling method was used. We measured various dimensions of the right ventricle in different views. The data was analyzed in the Statistical Package for the Social Sciences.

**Results:**

In the 96 male students included in the study, the mean right ventricular basal diameter was 36.45±3.49 mm, right ventricular mid cavity diameter was 29±3.63 mm, right ventricular longitudinal dimension was 65.72±7.52 mm, right ventricular outflow tract in parasternal long-axis view was 27.07±2.12 mm, proximal and distal right ventricular outflow in parasternal short-axis view was 25.33±2.57 mm and 20.08±1.99 mm, right ventricular thickness was 4.20±0.54 mm, and tricuspid annular plane systolic excursion was 23.02±3.54 mm.

**Conclusions:**

The study found that the values of right ventricular dimensions and the right ventricle's tricuspid annular plane systolic excursion among male medical students of a tertiary care hospital to be in accordance with the guidelines by the American Society of Echocardiography. The upper limits of the normal values of the right ventricle could be very helpful in clinical practice in determining the right ventricle dimension.

## INTRODUCTION

The right ventricle (RV) is the most anteriorly situated cardiac chamber, which is located behind the sternum.^1^ It is crescent-shaped in a cross-sectional view and triangular shape when viewed from the front with irregular endocardial surfaces and complex contraction mechanism.^1,2^

Recently, the interest in the evaluation of right ventricular size and function has increased significantly with newer echocardiographic techniques and a better understanding of the many medical conditions affecting the right heart.^3^ There are limited data on the normal dimension of the RV.^4^ The assessment of its size is important in diseases associated with right ventricular enlargement. According to the recent American guidelines,^5^ comprehensive examinations of the RV should be routinely performed.

There is no study done on the Nepalese to measure the normal values of the RV. The study aimed to measure right ventricular size, its thickness, and tricuspid annular plane systolic excursion in healthy male medical students of Kathmandu Medical College and Teaching Hospital.

## METHODS

This is a descriptive cross-sectional study done among healthy medical students of Kathmandu Medical College and Teaching Hospital, Kathmandu, Nepal. The study was conducted from February to April 2019. The ethical approval was taken from the Institutional Review Committee of the Kathmandu Medical College and Teaching Hospital (ref no. 120720193). The healthy volunteer male medical students with normal echocardiograms were included in the study. The female medical students and participants with abnormal echocardio-graphic findings, chronic medical illness, and ones with abnormal chest and thoracic deformities were excluded from the study. Informed written consent was taken from the participants. Convenient sampling was done. The sample size was calculated by using the formula,

$$\begin{array}{ll} \text{n} & \text{= }{\text{Z}}^{\text{2}}\times \text{p}\times \text{q}/{\text{e}}^{\text{2}}\\ & \text{= }{\left(1.96\right)}^{\text{2}}\times (0.5)\times (1-0.5)/{(\text{0.10)}}^{\text{2}}\\ & \text{= }96 \end{array}$$

where,

n = sample size,
Z = 1.96 at 95% Confidence Interval,
p = anticipated population proportion, 0.5
q = 1-p,
e = margin of error, 10%

We asked participants questions regarding preliminary data on demographics and medical history. The systemic examinations were done to rule out the presence of any disease. Weight in kilogram, height in centimeters, body mass index in kg/m^2^ and body surface area in square meter were recorded.

The comprehensive Echocardiographic examination was carried out for the measurement of right ventricle sizes, its thickness, and tricuspid annular plane systolic excursion (TAPSE). The different diameters of the right ventricle were measured in different views at the end of the diastole. The RV dimension was measured from a right ventricle-focused apical four-chamber view at the end of the diastole. The right ventricle basal diameter was measured in the basal one-third of the ventricular cavity.^6,7^ The RV mid cavity was measured in the middle third of the RV at the level of the RV papillary muscles. The RV longitudinal dimension was measured from the plane of the tricuspid annulus to the right ventricle apex.^5^

The proximal right ventricular outflow tract (RVOT) was measured in the parasternal long-axis view (PLAX). In the parasternal short-axis view (PSAX), the proximal right ventricular outflow tract was measured from the anterior aortic wall to the right ventricle free wall above the aortic wall and the distal RVOT was measured just proximal to the pulmonary artery.^5^

RV free wall thickness was measured at end-diastole by two-dimensional echocardiography from the subcostal window at the level of the tip of the anterior tricuspid leaflet.^5^ Parameters indicating RV function like TAPSE was acquired by placing an M-mode cursor through the tricuspid lateral annulus and measuring the amount of longitudinal motion of the annulus at peak systole in the apical 4 chamber view.^5^ The report of the measurement was recorded. The data were entered into Microsoft Excel and analyzed in Statistical Package for the Social Sciences (SPSS) version 20. Right ventricular morphological and functional measurements were expressed as means±standard deviation, and range.

## RESULTS

Among 96 medical students, the minimum and maximum age was 20 and 29 years respectively with a mean age of 24.71 ± 1.62 years. The body surface area was commuted from the height and weight of the patient using the Dubois Formula.^8^ The body surface area ranged from 1.48 to 2.07 m^2^ with a mean of 1.79 m^2^. The right ventricle mid diameter was 29.0±3.6 mm, right ventricle thickness was 4.20±0.5 mm, and tricuspid annular plane systolic excursion was 23.02±3.54 mm (Table 1).

**Table 1:** Right ventricle dimensions, and TAPSE, in mm (n = 96).

Variables	Mean±S.D.	Range
RV Basal Diameter	36.40±3.40	24.0-42.0
RV Mid diameter	29.0±3.6	20.0-37.7
RV Longitudinal dimension	65.72±7.5	27.9-82.0
PLAX RVOT	27.0±2.1	20.0-31.70
PSAX Proximal diameter	25.3±2.6	20.0-32.2
PSAX Distal diam­eter	20.1±2.0	17.0-25.7
RV Thickness	4.20±0.5	2.2-5.7
TAPSE	23.02±3.54	36.0-17.0

The absolute measurement of the right ventricles obtained was normalized according to BSA.

The right ventricle basal diameter was 20.38±2.02 mm, the mean right ventricular basal diameter was 36.40±3.40 mm, the right ventricle thickness was 2.36±0.3 mm and the tricuspid annular plane systolic excursion was 12.88±1.98 mm after corrected for body surface area (Table 2).

**Table 2:** Dimensions of right ventricle and TAPSE, in mm/m^2^ corrected for BSA (n = 96).

Variables	Mean±S.D.	Range
RV Basal Diameter	20.38±2.02	12.39-25.15
RV Mid Diameter	16.47±3.08	10.84-38.75
RV Long Dimension	36.83±4.39	16.82-47.22
PLAX RVOT	15.16±1.36	11.75-19.09
PSAX Proximal Diameter	14.20±1.65	10.88-19.03
PSAX Distal Diameter	11.26±1.32	18.70-16.23
RV Thickness	2.36±0.32	1.35-3.21
TAPSE	12.88±1.98	9.67-19.42

## DISCUSSION

The study measured the RV dimensions and function in terms of TAPSE (tricuspid annular plane systolic excursion ) among medical students with normal echocardiography.^9^ The right ventricular size and function are affected in many disease conditions that directly affect the musculature of the RV or indirectly from the LV.^9^ So, the evaluation of the RV should be part of every routine echocardiographic examination.^5^ In our study, echocardiography was prompted to get a range of normal values of different right ventricular internal dimensions, its thickness, and tricuspid annular plane systolic excursion as a function of the RV.

The absolute measurement of RV size in our study is comparable to other studies. The absolute size of the right ventricular basal diameter found in our study was 36.40±3.40 mm with a range of 24.0-42 mm which was comparable with a study done among top-level athletes done by D'Andrea, et al.^10^ and after corrected for body surface area were 20.38±2.02 with a range of 12.39-25.15. Similarly, the right ventricular mid cavity diameter and right ventricular longitudinal dimensions were 29.0±3.6 mm and 16.47±3.08 with a range of 20.0-37.7 and 10.84-38.75 and after corrected for body surface area were 16.47±3.08 mm, 36.83±4.39 mm, with a range of 10.84-38.75 mm, 16.82-47.22 mm.

The result obtained in our study is like the guidelines for the echocardiographic assessment of the right heart in adults by the American society of echocardiography. The morphological estimates got in the guidelines for normal right ventricular basal diameter, right ventricular mid cavity diameter, and right ventricular longitudinal dimensions mean and 95% CI were 33 (31-35) mm, 28 (23-33) mm and 71 (67-75) mm and upper reference values were 42 mm, 35 mm and 86 mm respectively at the end of the diastole.^5,11^

The proximal portion of the right ventricular outflow tract in the parasternal long-axis view with a mean of 27±2.1, range of 20-31.7, and after correcting for body surface area were 15.16±1.36, range of 11.7519.09. These data are in accordance with recent guidelines for the echocardiographic assessment of the right heart in which proximal portion of right ventricular outflow tract in parasternal long-axis view with mean of 25 mm, range 23-27 mm and upper reference value 33 mm.^5,11^

The proximal and distal right ventricular outflow tract in PSAX view with mean of 25.3±2.6 mm, 20.1±2.0 mm, range 20-32.2 mm, 17-25.7 mm, and after correction for body surface area mean 14.20±1.65 mm, 11.26 ± 1.32, range 10.88-19.03 mm, 16.23-18.70 mm. The proximal right ventricular outflow tract was more than the values in Tibetians, as found by Yang, et al. in a study which compared echocardiographic parameters between healthy highlanders in Tibet and lowlanders in Beijing.^12^

The mean right ventricular thickness was 4.20±0.5 mm comparable to the study by Yang, et al.^12^ but slightly greater than the thickness of top-level athletes as reported by D'Andrea, et al.^10^ Individual values range up to, 5.7 mm. Even after correction for body surface area mean thickness of the right ventricle was 2.36±0.32 mm, range 1.35-3.21 mm. The mean thickness of the right ventricle at the level of the tricuspid valve was similar to findings by Foale, et al. Tricuspid annular plane systolic excursion represents a longitudinal function of the right ventricle.^13^ The mean of tricuspid annular plane systolic excursion found in the study was 23.02±3.54 mm, the range was 17-36 mm, and after corrected for body surface area 12.88±1.98 mm, range 9.67-19.42 mm. The mean was comparable to the study by Rudski, et al.^5^ and D'Andrea, et al.^10^ and the corrected mean was less than the findings by Yang, et al.^12^

There were limitations in this study as we conducted the study only in male medical students of a single institution. We need studies of healthy people of different ethnic and geographical regions to find accurate dimensions.

## CONCLUSIONS

The study finds the values of right ventricular size and it’s a tricuspid annular plane systolic excursion among male medical students of a tertiary care hospital to be in accordance with the guidelines by the American Society of Echocardiography. The upper limits of the normal values of the right ventricle could be very helpful in clinical practice. The anthropometric measurement like body surface was an important determinant of right ventricle dimension measurement.

## Conflict of Interest

**None.**
